# Development of an HPTLC-based dynamic reference standard for the analysis of complex natural products using Jarrah honey as test sample

**DOI:** 10.1371/journal.pone.0254857

**Published:** 2021-07-20

**Authors:** Md Khairul Islam, Tomislav Sostaric, Lee Yong Lim, Katherine Hammer, Cornelia Locher

**Affiliations:** 1 Cooperative Research Centre for Honey Bee Products Limited (CRC HBP), University of Western Australia, Perth, Western Australia, Australia; 2 Division of Pharmacy, School of Allied Health, University of Western Australia, Crawley, Western Australia, Australia; 3 School of Biomedical Sciences, University of Western Australia, Crawley, Western Australia, Australia; Institute for Biological Research, SERBIA

## Abstract

In this paper, we describe a novel approach to the development of a reference standard for the quality control of complex natural products, which will assist in the assessment of their authenticity and purity. The proposed method provides a template for the selection of samples, which can be pooled to obtain a reference standard. A shortfall of such an approach is, however, that the pooled sample is static in nature and therefore unable to capture difference in processing conditions or natural variations triggered by geographical or climatic impacts over time. To address this, the paper also outlines the development of a dynamic reference standard, which allows for ongoing adjustments to future variations. The method employs High-Performance Thin Layer Chromatography (HPTLC) derived extract profiles processed by multivariate analysis. The development of the dynamic reference standard is illustrated using honey, a complex natural matrix, as an example.

## Introduction

Natural products of plant and animal origin have been investigated physically, chemically or organoleptically for thousands of years [1, 2]. In particular for those that are used as medicinal, food or flavouring agents, a consistent phytochemical profile and with this predictability and reliability in appearance, taste, smell and also bioactivity are paramount [3–6]. The development of appropriate reference standards has therefore been a focus of quality control efforts in order to assess the authenticity, purity and potency of natural products. Many quality assurance methods for natural products rely on the qualitative and / or quantitative analysis of meaningful marker compounds [7–9]. Standardisation of these marker compounds, however, poses its own challenges, for example when the natural product’s bioactivity levels (along with its organoleptic characteristics) cannot easily be tied to a single or a few compounds [10] but are the result of a complex interplay of a variety of constituents [11–13], or in cases where key constituents have not yet been chemically identified [14]. The use of profile chromatograms, which reflect a natural product’s typical phytochemical composition, is therefore a common approach [15]. Furthermore, seasonal and geographical variations introduce an additional layer of complexity for the authentication and quality control of complex natural matrices. Rather than evaluating an extract against a profile chromatogram derived from a single reference sample of the natural product, pooling samples might therefore be more appropriate [16–18]. In this approach a number of samples, deemed to adequately represent the natural product extract, even across seasons or a wider geographical spread, are blended to create a pooled reference sample. Although there will necessarily be some variations between individual samples within the blend, the overall ‘picture’ that emerges from this pooled reference sample and its associated profile chromatogram will capture the extract’s typical phytochemical characteristics; pooling will ‘dilute out’ unusual constituents and amplify those that are common across the samples and so facilitate adequate quality control of complex natural products.

Two core challenges remain nonetheless. First, how to select samples for inclusion in this pooled reference sample and second, how to ensure that the pooled reference standard remains ‘current’ and continues to adequately reflect a natural product extract, which might change over time due to a range of external factors (e.g. climate change, change in growing or processing condition).

In this paper, we describe a novel method for the development of a reference standard, which is able to address both of these challenges. It provides a template for the selection of samples and, due to its dynamic nature, also allows for ongoing adjustments to future variations. The approach is based on High-Performance Thin Layer Chromatography (HPTLC) extract profiles and their multivariate analysis. The development of such a dynamic reference standard is illustrated using honey, which is a complex natural matrix, as an example.

The phytochemical composition of honey, and with this its organoleptic and bioactivity profile, is directly related to its floral origin [19–21], namely the flowers bees visit and the nectar they collect. Variations in composition are reflected in a honey’s organic extract and can be captured in the respective HPTLC profile [22–24]. Monofloral honeys, which are predominately derived from a single floral source, are highly sought after and priced accordingly [25, 26]. However, wild harvested honeys are never 100% monofloral in their origin as bees cannot be restricted to a particular foraging area and will collect nectar opportunistically based on preferences and nutritional needs [27, 28]. Furthermore, geographical and environmental variations of nectar producing plants, differences in harvesting methods and post-harvest processing as well as variations in storage conditions can also impact on the final phytochemical composition of honey [29–31]. This complexity makes honey an ideal model natural product to investigate the development of a dynamic reference standard for authentication and quality control.

Specifically, this study focuses on Jarrah honey, which originates from *Eucalyptus marginata*, a tree endemic to the south west of Western Australia [32]. This highly antibacterial and antioxidant honey [33] is mainly harvested from native forests and nature reserves, which means that Jarrah honeys might be mixed with other floral sources. To ensure a quality product, the authentication of the honey’s predominant nectar source against a representative reference standard is therefore an important undertaking. Over the years a range of authentication methods for honey have been developed [34]. For example, melissopalynology is commonly used to authenticate honeys, although in the specific context of Australian eucalypt honeys this approach is not without its challenges [35–39]. The method introduced in this paper outlines how a dynamic reference standard can be developed based on the multivariate analysis of HPTLC profiles of Jarrah honey organic extracts, which might then be used as an alternative authentication tool.

It needs to be emphasised that the combination of HPTLC and multivariate analysis is not new, not even in the context of honey analysis [24, 40–42]. What is novel about this study is the way, outlined below, how multivariate analysis of HPTLC profiles can be used to derive a dynamic, representative reference standard for quality control purposes. Two steps are proposed to derive this reference standard:

1. Identify similarities in the HPTLC profile to determine the *target cluster*, which constitutes an aggregation of samples with similar constituent profiles. This step assists in defining the target cluster by removing extreme outliers as well as samples that are of multifloral origin.
2. Additional screening of the target cluster to remove diluted samples as well as those that have moderate quantities of other floral sources present. This step assists with the definition of the *core cluster*.

In the first step, the HPTLC fingerprints of the target samples are analysed alongside other samples of a different floral origin and thus with different HPTLC fingerprints. This assists in *defining target cluster characteristics* common to all target samples by discriminating from samples with different HPTLC profiles. Thus, this initially identified target cluster will only contain samples with similar characteristics; extreme outliers or samples of multifloral origin will be removed from the target cluster at this stage. The samples constituting this target cluster are then carried forward into the second analysis step.

In the second step, samples forming the target cluster will be analysed again. As in this analysis round no additional samples are introduced, the target cluster will be refined with dilute samples and those that contain moderate quantities of other floral origins shifting towards the periphery of the cluster. This will allow to identify those *samples that constitute the core cluster*. Reiterations of the second step are possible in order to further refine the core cluster.

Samples in the identified core cluster can then be pooled and the blend be used as a reference tool for quality control purposes as the representative fingerprint and chromatographic profile of the combined sample will capture all the characteristics and natural variations of the natural product extract. However, such a physical reference sample is static in nature and might no longer be able to capture and reflect externally driven changes (e.g. change in growing or processing conditions, climate change). An alternative to this pooling approach is therefore to work with a dynamic reference sample, which continues to reflect even subtle changes to the natural product extract’s phytochemical composition over time, in fluent, or dynamic, cluster boundaries.

Using Jarrah honey as case example, we will explore the various steps leading to the identification of the target and core clusters and then assess three Jarrah honeys against the pooled reference sample as well as the dynamic reference standard, which were both developed on the basis of multivariate analysis, in order to determine their floral authenticity.

## Materials and methods

### Reagents, chemicals and samples

All reagents and chemicals used in this study were of analytical grade. Honey samples (n = 104) were collected from beekeepers of Western Australia (Table 1). They were classified according to the information provided on the label and no further initial tests were carried out to confirm their floral identity.

**Table 1:** Honey samples including codes, common name, botanical source and number of samples.

Codes	Common Name ^a^	Botanical Source ^b^	Number of Samples
MAR	Marri (Redgum)	*Corymbia calophylla*	1
KAR	Karri	*Eucalyptus diversicolor*	1
JAR	Jarrah	*Eucalyptus marginata*	46
WAN	Wandoo	*Eucalyptus wandoo*	1
BAS	Parrotbush	*Banksia sessilis*	14
BAM	Banksia menziesii	*Banksia menziesii*	10
GOL	Goldfields	Mixed floral	1
RED	Red Bell	*Calothamnus quadrifidus*	1
WHI	Whitegum	Whitegum sp.	1
LEP	Leptospermum	*Leptospermum spp*.	1
YAT	Yate	*Eucalyptus cornuta*	1
POW	Powderbark	*Eucalyptus accedens*	1
BRO	Brown Mallet	*Eucalyptus astringens*	1
CAL	Callistemon	*Callistemon spp*.	1
MOO	Moort	*Eucalyptus platypus*	1
WIL	Wildflower	Mixed floral	1
BAN	Banksia	*Banksia spp*.	4
ERE	Eremophila	*Eremophila spp*.	1
MAL	Mallee	Mixed floral	1
SPR	Spring	Mixed floral	1
CAN	Canola	*Brassica napus*	1
CAP	Capeweed	*Arctotheca calendula*	1
EUC	Great Victoria Desert	Mixed floral	1
MEL	Melaleuca	*Melaleuca alsophila*	1
SCH	Scholtzia	*Scholtzia spp*.	1
ACA	Acacia	*Acacia spp*.	1
BAP	Banksia prionotes	*Banksia prionotes*	1
BAV	Orange Banksia	*Banksia victoriae*	1
BAG	Banksia (Bull)	*Banksia grandis*	1
BLO	Bloodwood	*Corymbia zygophylla*	1
GIM	Gimlet	*Eucalyptus salubris*	1
ORA	Orange Blossom	*Citrus X sinensis*	1
TAG	Tagasaste	*Cytisus proliferus*	1
WAT	Watermelon	*Citrullus lanatus*	1
Total			104

^a^ Common name as provided by beekeepers.

^b^ Botanical source as determined by beekeepers.

To obtain the organic honey extracts for HPTLC fingerprinting, 1 g of each honey was dissolved in 2 ml of deionised water. The aqueous honey solution was extracted three times with 5 ml of dichloromethane. After drying with anhydrous MgSO_4_ the combined organic extracts were evaporated at ambient temperature and stored at 4°C until further analysis.

### High-Performance Thin Layer Chromatography (HPTLC) fingerprinting

To obtain the honey extracts’ respective chromatographic profiles, the method described by Locher et al. [22, 23] was followed. In brief, after reconstituting in 100 μL of dichloromethane the organic extracts were analysed by HPTLC (CAMAG, Muttenz, Switzerland). For this 5 μL of each extract were applied to the HPTLC plates (silica gel 60 F_*254*_ HPTLC glass plates, 20 x 10 cm), which were developed at ambient temperature to a final solvent migration distance of 70 mm using toluene: ethyl acetate: formic acid (6:5:1, *v/v/v*) as mobile phase. After drying, the plates were analysed at 254 nm and 366 nm (TLC Visualiser 2). The plates were then derivatised using vanillin spraying reagent and heated for 3 min at 115°C (CAMAG TLC Plate Heater III, Muttenz, Switzerland). Finally, the cooled plates were analysed at white light and 366 nm.

### Data acquisition

**Fig 1:**
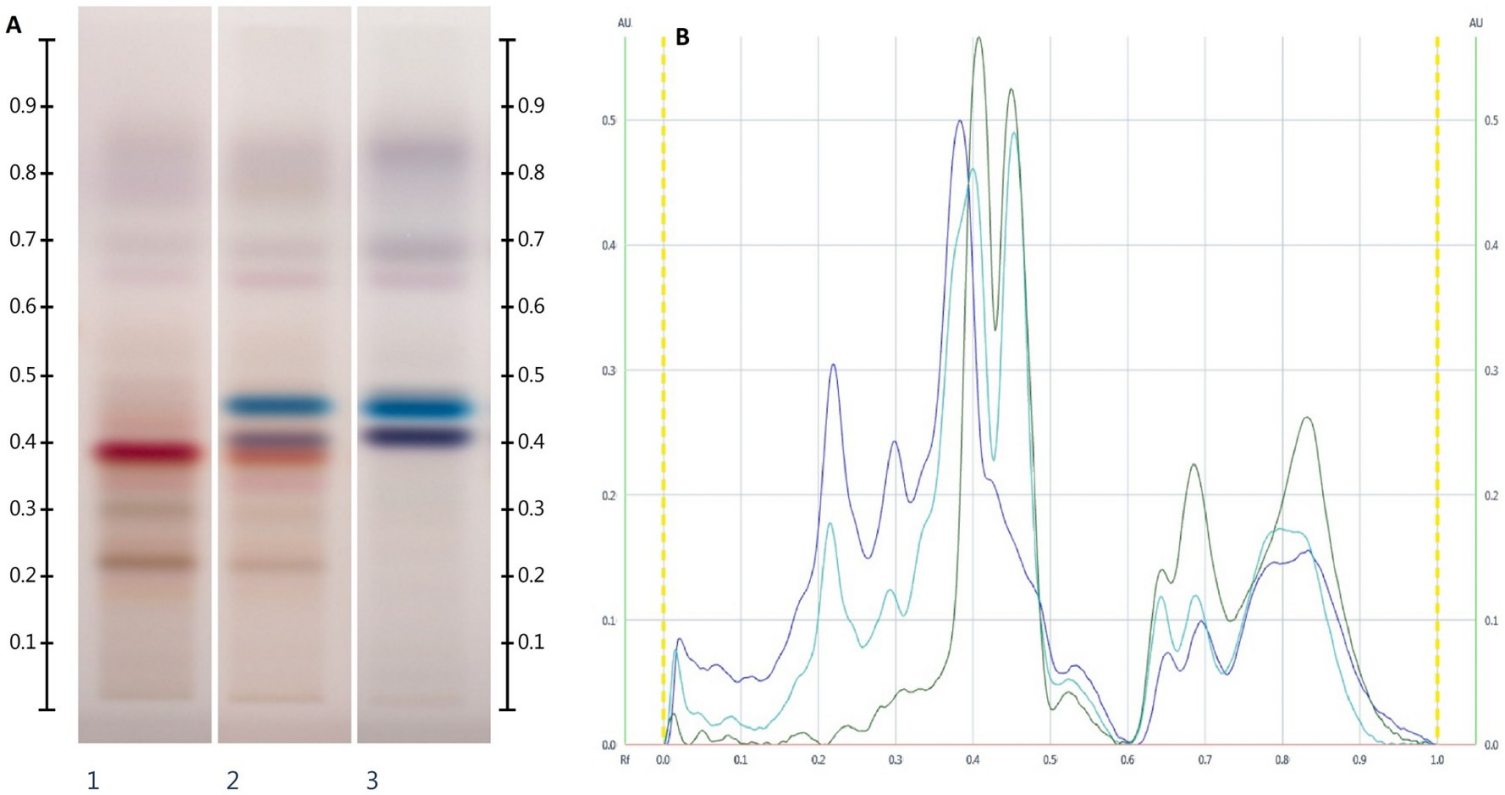
Images taken at White light after derivatisation with vanillin reagent; Track 1 –JAR, Track 2 –multifloral honey (JAR mixed with BAS), Track 3 –BAS (a), and their respective chromatograms (b); 5 μL of each extract respectively.

The four sets of fingerprints of each sample (at 254 nm and 366 nm, and at white light and 366 nm after derivatisation) were converted into their respective chromatograms to derive values for migration distances (Rf) and corresponding intensities (AU). Furthermore, the colour (as RGB values) of the corresponding HPTLC bands was also recorded. Only bands with a Rf value between 0.05 and 0.60 were considered as this captured the majority of bands. While some additional bands with higher Rf values can be seen in the respective HPTLC fingerprint (Fig 1) they are not specific to a honey’s floral source and thus will not contribute any important bands towards the development of the reference standard. Given the lipophilic nature of the extraction solvent, these bands most likely represent waxy honey constituents, which are more reflective of the honey’s processing conditions (e.g. to what extent filtration was used to remove waxes and other debris from raw honey) rather than its floral source. For the ensuing two-step multivariate analysis, band intensities (AU) were multiplied with their corresponding RGB values (as red, green and blue pixels) and the result plotted against the corresponding Rf values. A 1248 x 104 data matrix was derived from this approach and multivariate analysis (Principal Component Analysis) was performed on this dataset using R and R Studio [43, 44].

## Results and discussion

### Visual assessment of fingerprints

The obtained HPTLC profiles of the various honey extracts consisted of four sets of fingerprints and their corresponding chromatograms. While the chromatograms hold two-dimensional information (Rf vs Intensity), the fingerprint itself is richer as the various bands also differ in colour. When placed side by side visual differences and communalities in the respective HPTLC fingerprints can be assessed. Fig 1a exemplifies the diversity in fingerprints prior to clustering.

This richness in data can be seen as one of the advantages of HPTLC analysis over other methods (e.g. HPLC) that can also be employed to derived chromatographic profiles. Individual compounds can be accounted for after development in two different conditions (254 and 366 nm) as well as after derivatisation, again in two different conditions (white light and 366 nm), with the different band colours captured in their respective RGB values. With this wealth of data even compounds with very similar or even identical chromatographic behaviour (i.e. Rf value) can be distinguished from each other.

### Cluster analysis

**Fig 2:**
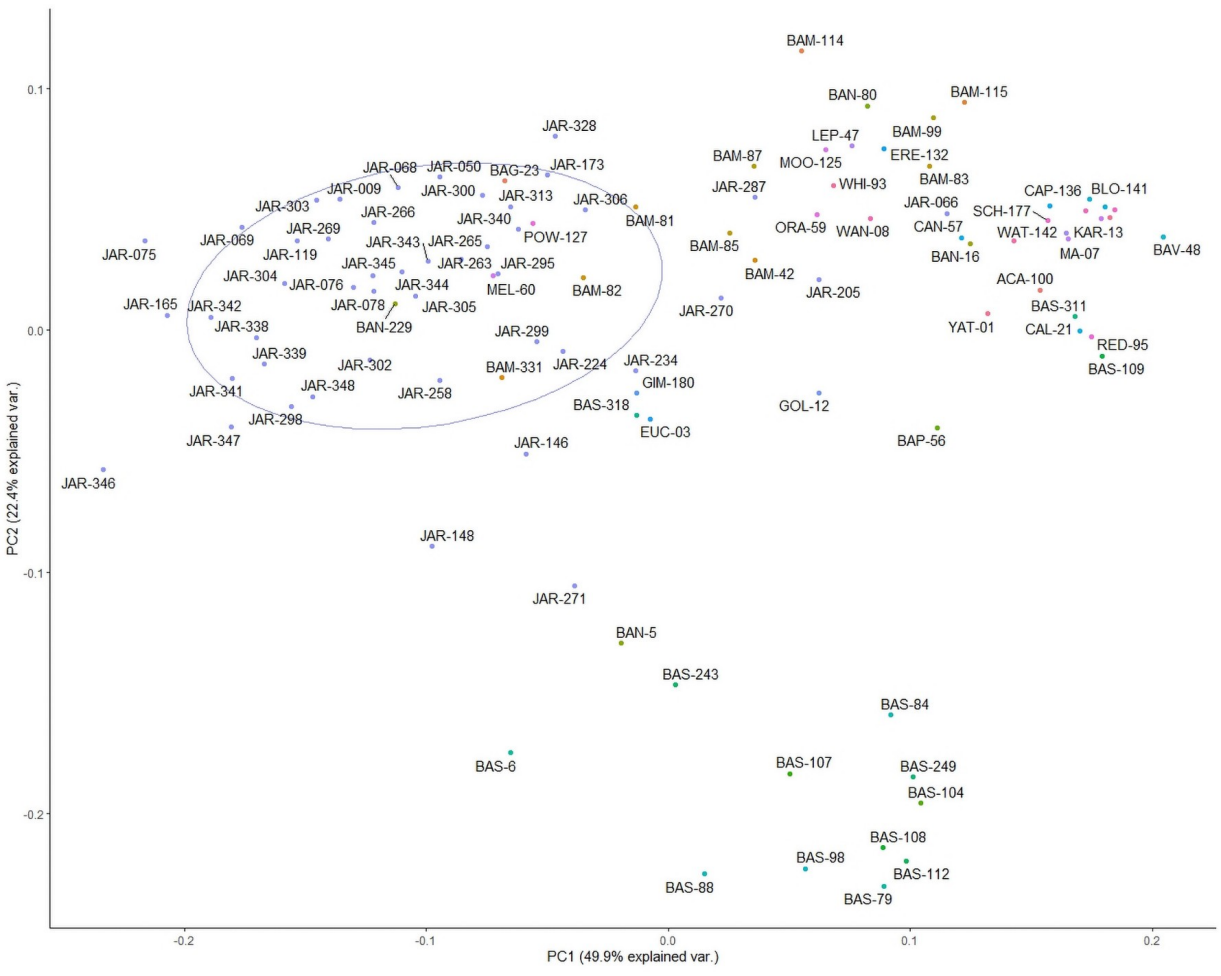
First step of cluster analysis (identification of JAR target cluster).

Unsupervised non targeted multivariate analysis (Principal Component Analysis) was conducted in order to position all samples in a simple cluster diagram. In the first step of the analysis, aimed at identifying the target cluster, the fingerprints of 104 honeys, were analysed. They included, as labelled by the beekeeper, 46 Jarrah honeys (JAR), 14 *Banksia sessilis* honeys (BAS), 10 *Banksia menziesii* honeys (BAM), 4 Banksia honeys from unidentified Banksia species (BAN) as well as 30 other honeys of 30 different floral origins (Table 1). The cluster diagram shown in Fig 2 demonstrates that several clusters formed in this first round of multivariate analysis and that the target cluster (JAR) is clearly separated from the rest of the analysed samples. There are some samples which are situated between the JAR target cluster and the BAS cluster and visual inspection of the fingerprints of those particular samples confirmed that they were of mixed floral origin. Similar observations were made for samples found between the JAR target cluster and the BAM cluster. Some samples, for instance BAG-23, POW-127, BAM-82, BAM-331, MEL-60 and BAN-229 (Fig 2), appeared within the JAR target cluster although they were not identified as Jarrah honeys by the beekeepers. Visual inspection of their corresponding HPTLC profiles confirmed that these were of multifloral origin with evidence of nectar sources other than Jarrah. On the other hand, some JAR samples (e.g. JAR-271, JAR-205) were found outside the JAR target cluster. In this case visual inspection of the respective HPTLC profiles confirmed that they were outliers and most likely misclassified by the beekeepers.

To prepare for the second step of the analysis, aimed at defining the JAR core cluster, a 60% probability circle was placed around the target cluster, which contained 38 samples representing the JAR target cluster.

**Fig 3:**
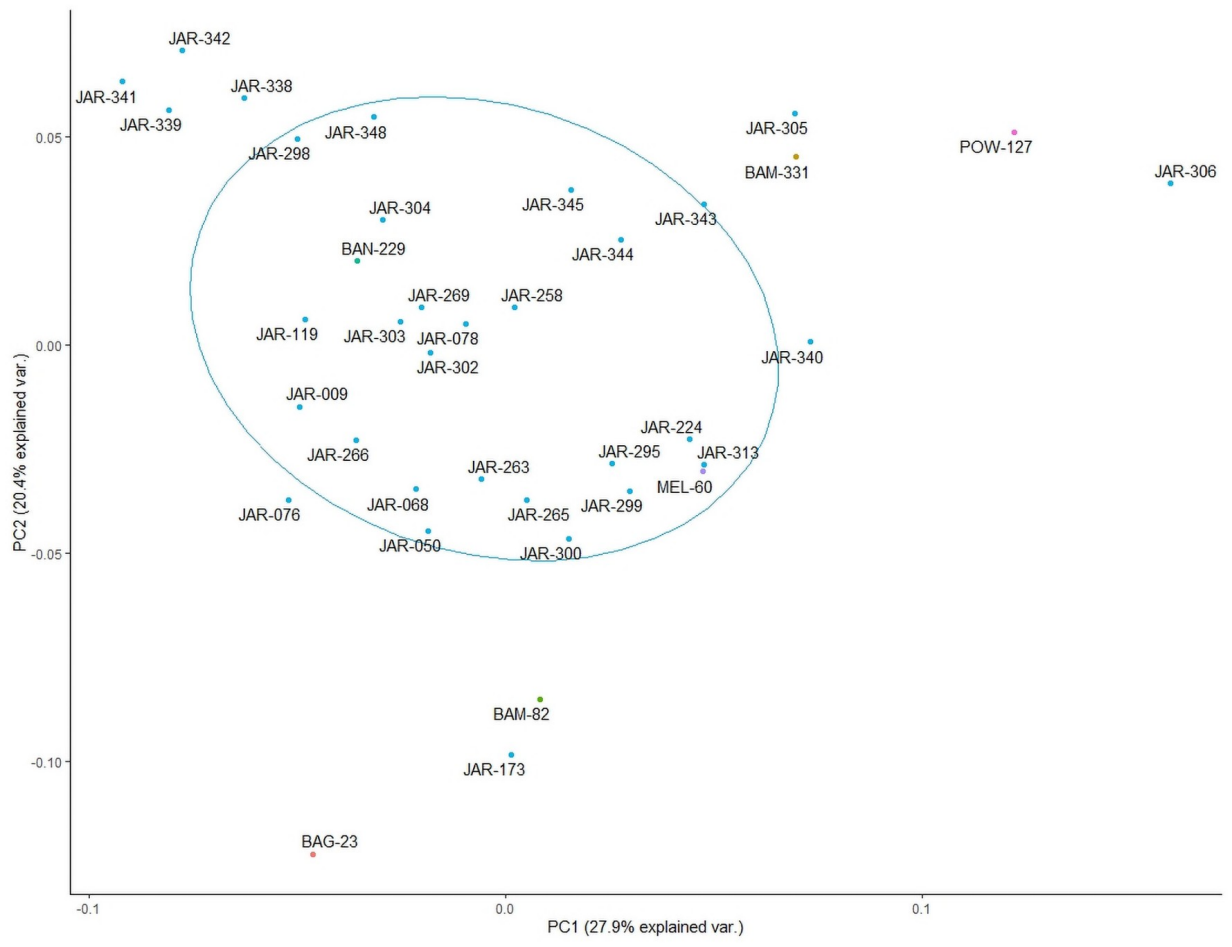
Second step of cluster analysis (identification of preliminary JAR core cluster).

In a second step the fingerprints of the 38 samples constituting the JAR target cluster were analysed again by multivariate analysis. Fig 3 illustrates that in this step the initial JAR target cluster was re-arranged to form a new cluster pattern where some samples (e.g. JAR 173, JAR 305) had shifted towards the periphery of the cluster. A visual inspection of their HPTLC profiles confirmed that those samples contained moderate quantities of other floral sources. Again, a 60% probability circle was placed around the cluster, which now contained 24 samples representing the preliminary JAR core cluster. Interestingly, two samples MEL-60 and BAN-229 were still found within the JAR core cluster. Visual inspection of their HPTLC profiles confirmed that their fingerprints were closely related to the typical fingerprint of JAR, which might indicate that their predominant floral origin was misclassified by the beekeepers.

**Fig 4:**
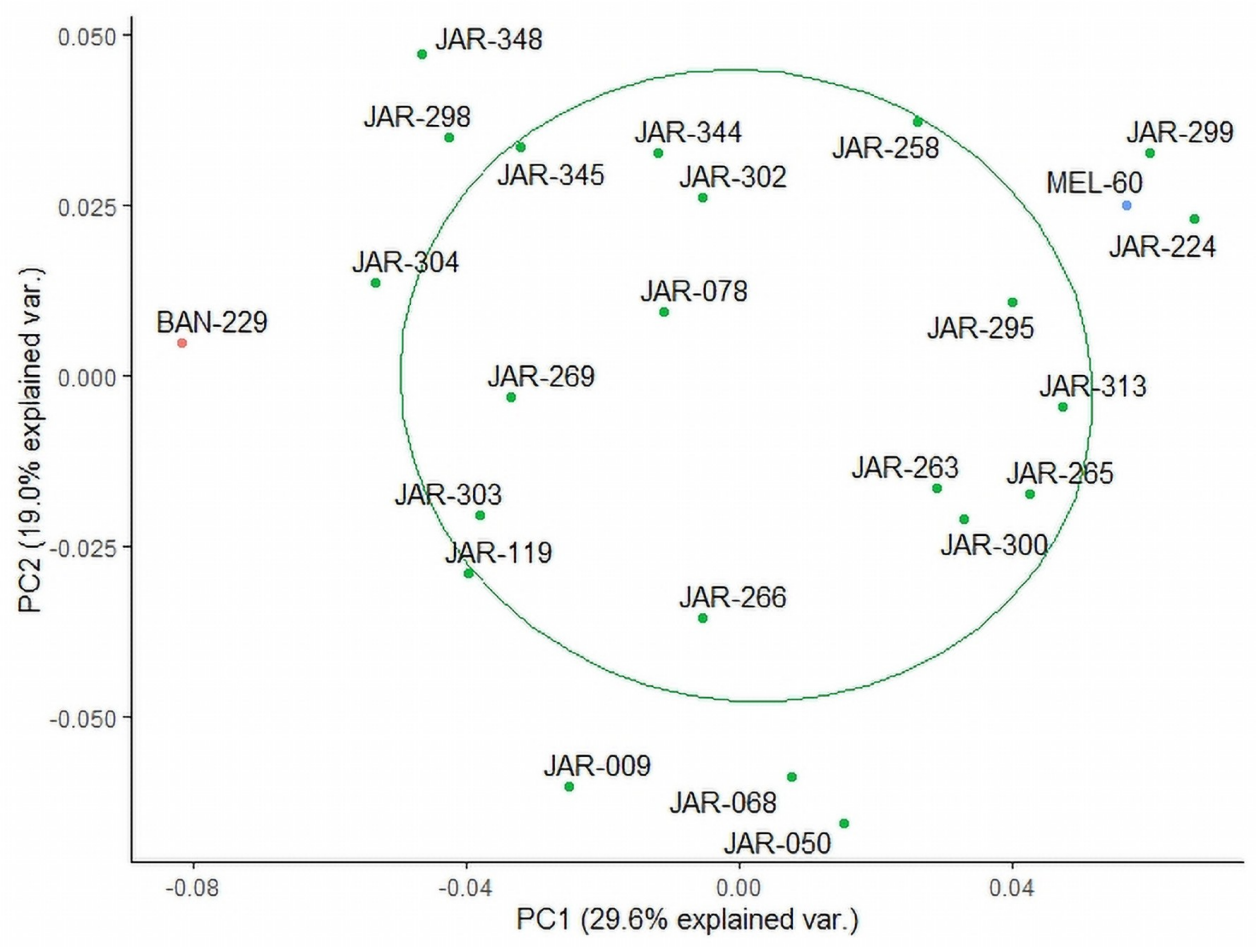
Final step of cluster analysis (identification of refined JAR core cluster).

In order to further refine the cluster, another multivariate analysis was performed on the 24 samples constituting the preliminary JAR core cluster. Fig 4 demonstrates that the samples in the preliminary JAR core cluster again re-arranged to form a new cluster pattern. Those samples, which were dilute JAR samples or contained minor quantities of other floral sources, shifted towards the periphery of this refined JAR core cluster, which was marked again with a 60% probability circle and contained 13 JAR samples. It is interesting to note that this refined JAR core cluster no longer contains MEL-60 and BAN-229. A visual inspection of the HPTLC profiles of the samples constituting this refined core cluster presented a consistent fingerprinting pattern (Rf values, colour) and band intensities (representing the respective compound concentrations) in all four HPTLC derived images (254 nm, 366 nm as well as 366 nm and white light after derivatisation).

We argue that these 13 JAR samples represent the characteristics of the refined JAR core cluster and therefore can be used to prepare a pooled sample in order to obtain a JAR reference standard for quality control purposes.

### Preparation of pooled reference standard

Equal amounts of the 13 JAR samples identified as representing the refined JAR core cluster were pooled and warmed to 37°C to assist with the preparation of a homogenous mixture. This mixture constitutes the JAR reference standard.

To obtain the HPTLC profile of this reference standard, 1 g of the mixture was dissolved in 2 ml of deionised water and the aqueous honey solution extracted three times with 5 ml of dichloromethane. The combined extracts were dried at ambient temperature and then analysed as described in the Methods section. The obtained fingerprints and associated chromatograms of this pooled sample extract could be used in honey quality control to confirm the floral identity of a sample claimed to be JAR honey.

### Development of a dynamic reference standard

As described in the following section in more detail, instead of preparing a physical pooled reference standard for quality control purposes, it is also possible to generate a dynamic reference standard from the collated data, based on the above described multivariate analysis. An advantage of this approach is that when clustering statistically, samples are not mixed physically, leaving a pathway for the future addition of new samples into the clustering process. Those samples might bring additional minor information, which might reflect changes in growing or processing conditions or climatic changes that impact on the phytochemical composition of the samples.

### Proof of concept: Confirmation of floral source of Jarrah honey samples using a dynamic reference standard

**Fig 5:**
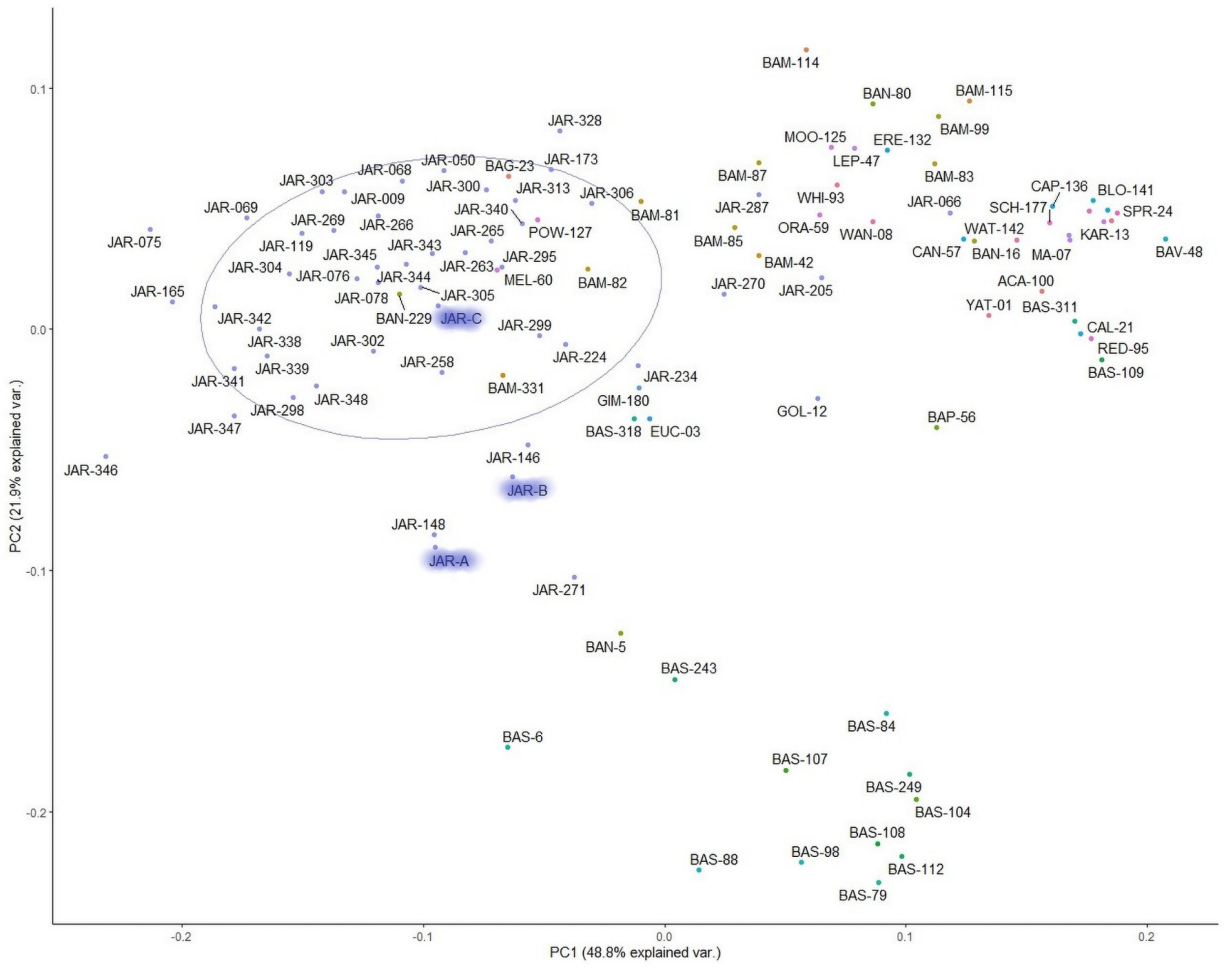
Cluster analysis with new Jarrah samples (JAR-A, JAR-B, JAR-C).

**Fig 6:**
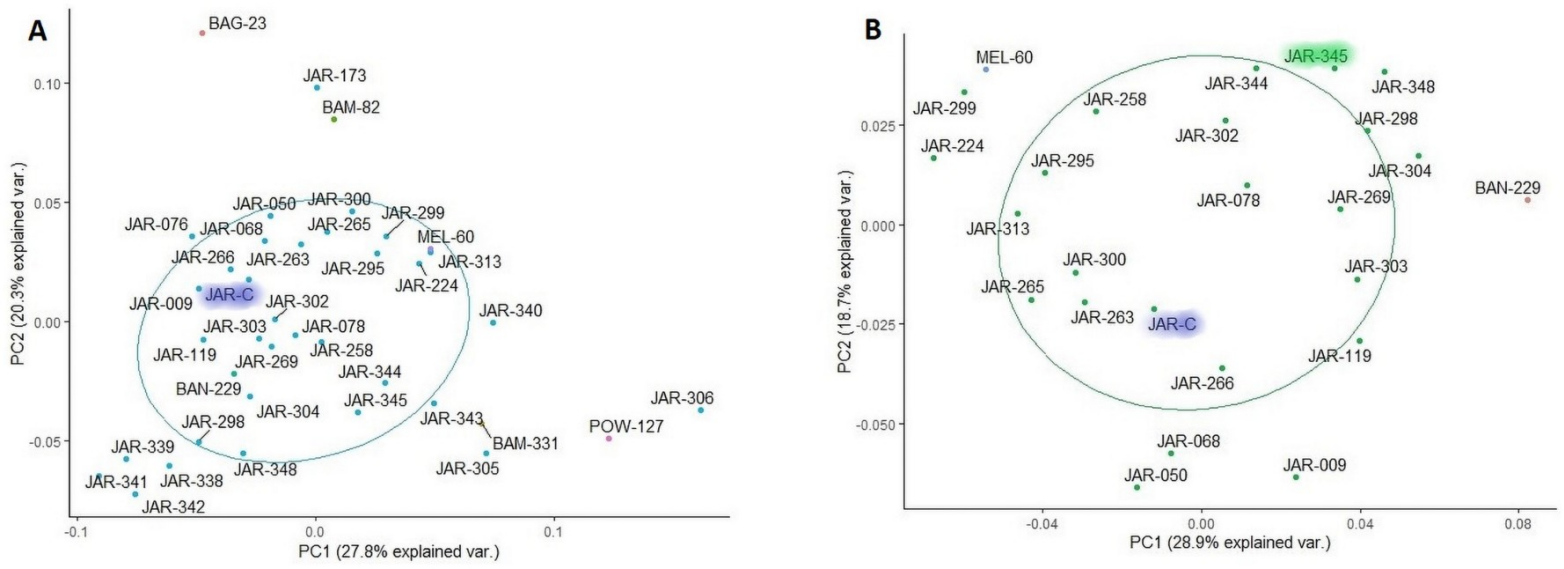
Cluster analysis with new Jarrah samples (JAR-A, JAR-B, JAR-C); preliminary JAR core cluster (a) and refined JAR core cluster (b).

Three honeys, declared by beekeepers as Jarrah honeys (here referred to as JAR-A, JAR-B and JAR-C) were extracted and HPTLC fingerprinted as described in the Methods section. The obtained information was then included in the data matrix and clustering performed on the new data set. JAR-C was found in a central position within the JAR target cluster, and subsequently also in the preliminary and refined core clusters (Figs 5, 6a and 6b) but the other two samples (JAR-A and JAR-B) were outside the target cluster’s boundary. Visual comparison of the HPTLC fingerprints obtained for the three samples with those of the 13 JAR samples previously identified as representing the refined JAR core cluster demonstrate very close agreement for JAR-C. While containing all the characteristic fingerprinting features of the 13 JAR refined core cluster samples, JAR-A and JAR-B, however, present additional bands, indicating that these two honeys contain mainly JAR nectar, but also small amounts of other floral sources.

As can be seen from Fig 6a and 6b, JAR-C not only clustered relatively central within the preliminary JAR core cluster, which contained a total of 39 samples, but also in the refined core cluster (25 samples). On this basis, JAR-C can be considered to represent an authenticated JAR honey, which thus can be added to the dynamic reference standard. A careful comparison between the refined JAR core cluster, obtained prior to the inclusion of JAR-C, and the new refined JAR core cluster (including JAR-C) illustrates the dynamic nature of the standard and its slightly fluid and evolving cluster boundaries. This can be seen in subtle shifts in the cluster position of some samples. For instance, sample JAR-345, which was included in the initial refined core cluster (Fig 4) has moved just outside the new core cluster’s 60% probability circle (Fig 6b). The slight shift is due to the addition of three new samples (JAR-A, JAR-B and JAR-C) in the analysis and ultimately the addition of a new sample (JAR-C) to the refined core cluster, which demonstrates the dynamic nature of the method.

It needs to be emphasised that the above dynamic reference is currently based on the analysis of only 49 JAR samples and contains only 13 JAR core cluster samples. With the addition of new samples in the future and continued multivariate analysis, the refined core cluster’s boundaries might shift slightly, capturing the dynamic nature of the reference standard. This will allow to reflect, much more easily than could be done with the use of a pooled reference standard, natural diversity triggered, for example, by environmental, geographical, seasonal or processing variations.

The paper also proposes a step-wise approach in determining the core cluster. How many iterations of the clustering will be carried out to ultimately derive at the final core cluster (i.e. in this study two iterations were carried out) is flexible and thus allows the method to be designed to be fit-for-purpose. The same applies to the decision at what level the cluster borders are set (i.e. in this study the probability circle was set at 60%). We envisage that this process requires stakeholder input (e.g. industry standards, internal quality control requirements) to determine how narrow or extended the cluster boundaries need to be in order to derive meaningful pooled or dynamic reference standards that meet stakeholder requirements. It might even be possible with this approach to determine samples of different quality. For instance, it might be decided that samples that fall into the target cluster meet the requirements of the respective Food Codex standard, whereas samples that are earmarked for use as (complementary) medicines and thus need to fulfil certain Monograph standards, will need to fall within the core cluster.

There are some limitations to the preparation of such a dynamic reference standard: An adequate number of target samples (here JAR) should be available to derive HPTLC fingerprints and corresponding chromatograms that adequately represent the cluster and the target samples should also be more numerous than ‘other’ samples that, from the onset, are outside the target cluster (here e.g. BAN, BAM, MEL–see Table 1). Furthermore, the target samples should be of an acceptable purity as a large number of highly contaminated or diluted samples might lead to a misrepresentation of the core cluster.

## Conclusion

Assessment of authenticity and purity of complex natural products is a challenging undertaking, in particular in cases where key bioactive constituents that could act as marker compounds for standardisation are not (yet) identified or where a complex interplay of a variety of key constituents characterises the extract. In such cases quality control commonly relies on profile chromatograms to adequately capture the complexity of the natural product. Given natural variations in composition, it is advisable to prepare such profile chromatograms from pooled reference samples. This paper outlined a new method for how samples for such a pooled reference can be selected on the basis of HPTLC fingerprinting followed by multivariate analysis. Employing the same analysis approach, the study also described the development of a dynamic reference standard as an alternative to a static pooled reference sample, which is able to better capture seasonal, geographical, environmental or processing variations. The approach was illustrated using Jarrah honey, but the developed method provides a template that can be also used for the preparation of quality control standards (pooled samples and / or dynamic references) of other natural products.

Where available, an initial single botanical reference material (BRM) can be included in the analysis as a starting point from which the dynamic reference standard can be developed. Further, while this study has based its multivariate analysis on HPTLC derived data, future research should explore if, in other contexts, a similar clustering can also be achieved using different analytical input data. Here, the analysis has been based on individual constituent bands’ Rf values, colour and peak intensities and it would be interesting to explore if a combination of constituents’ retention times, peak areas and spectral data derived by High Pressure Liquid Chromatography (HPLC), for example, might provide equivalent discrimination power.

## Supporting information

S1 File Clustering file. (CSV)

## References

[1] Marston A, Hostettmann K. Natural product analysis over the last decades. Planta Med. 2009;75(7):672–82. Epub 2009/03/06. doi: 10.1055/s-0029-1185379 . 19263341

[2] Cragg GM, Newman DJ. Natural products: A continuing source of novel drug leads. Biochimica et Biophysica Acta (BBA)—General Subjects. 2013;1830(6):3670–95. doi: 10.1016/j.bbagen.2013.02.008 23428572 PMC3672862

[3] Schröder MJA. Origins and Nature of Sensory and other Performance Attributes in Foods. Food Quality and Consumer Value: Springer; 2003. p. 137–65.

[4] Atanasov AG, Waltenberger B, Pferschy-Wenzig E-M, Linder T, Wawrosch C, Uhrin P, et al. Discovery and resupply of pharmacologically active plant-derived natural products: A review. Biotechnol Adv. 2015;33(8):1582–614. doi: 10.1016/j.biotechadv.2015.08.001 26281720 PMC4748402

[5] Upton R, David B, Gafner S, Glasl S. Botanical ingredient identification and quality assessment: strengths and limitations of analytical techniques. Phytochem Rev. 2020;19(5):1157–77. doi: 10.1007/s11101-019-09625-z Upton2020.

[6] Simmler C, Graham JG, Chen S-N, Pauli GF. Integrated analytical assets aid botanical authenticity and adulteration management. Fitoterapia. 2018;129:401–14. doi: 10.1016/j.fitote.2017.11.017 29175549 PMC5963993

[7] Lazarowych NJ, Pekos P. Use of Fingerprinting and Marker Compounds for Identification and Standardization of Botanical Drugs: Strategies for Applying Pharmaceutical HPLC Analysis to Herbal Products. Drug Inf J. 1998;32(2):497–512. doi: 10.1177/009286159803200222

[8] Li S, Han Q, Qiao C, Song J, Lung Cheng C, Xu H. Chemical markers for the quality control of herbal medicines: an overview. Chin Med. 2008;3:7-. doi: 10.1186/1749-8546-3-7 18588699 PMC2488332

[9] Organization WH. WHO Expert Committee on Specifications for Pharmaceutical Preparations: Fiftieth Report: World Health Organization; 2016. 27514184

[10] Ruiz GG, Nelson EO, Kozin AF, Turner TC, Waters RF, Langland JO. A Lack of Bioactive Predictability for Marker Compounds Commonly Used for Herbal Medicine Standardization. PLoS One. 2016;11(7):e0159857. doi: 10.1371/journal.pone.0159857 27458926 PMC4961437

[11] Yang W, Zhang Y, Wu W, Huang L, Guo D, Liu C. Approaches to establish Q-markers for the quality standards of traditional Chinese medicines. Acta Pharmaceutica Sinica B. 2017;7(4):439–46. doi: 10.1016/j.apsb.2017.04.012 28752028 PMC5518652

[12] Ding M, Jiang Y, Yu X, Zhang D, Li J, Wang H, et al. Screening of Combinatorial Quality Markers for Natural Products by Metabolomics Coupled With Chemometrics. A Case Study on Pollen Typhae. Front Pharmacol. 2018;9(691). doi: 10.3389/fphar.2018.00691 30002628 PMC6033115

[13] Liang Y-Z, Xie P, Chan K. Quality control of herbal medicines. Journal of Chromatography B. 2004;812(1):53–70. doi: 10.1016/j.jchromb.2004.08.041 15556488

[14] Zeng Z, Chau F-t, Chan H-y, Cheung C-y, Lau T-y, Wei S, et al. Recent advances in the compound-oriented and pattern-oriented approaches to the quality control of herbal medicines. Chin Med. 2008;3(1):9. doi: 10.1186/1749-8546-3-9 Zeng2008. 18680568 PMC2531114

[15] Cieśla Ł, Moaddel R. Comparison of analytical techniques for the identification of bioactive compounds from natural products. Nat Prod Rep. 2016;33(10):1131–45. Epub 2016/07/02. doi: 10.1039/c6np00016a . 27367973 PMC5042860

[16] Salomé-Abarca LF, van der Pas J, Kim HK, van Uffelen GA, Klinkhamer PGL, Choi YH. Metabolic discrimination of pine resins using multiple analytical platforms. Phytochemistry. 2018;155:37–44. Epub 2018/08/03. doi: 10.1016/j.phytochem.2018.07.011 . 30071382

[17] Shewiyo DH, Kaale E, Risha PG, Dejaegher B, Smeyers-Verbeke J, Heyden YV. HPTLC methods to assay active ingredients in pharmaceutical formulations: A review of the method development and validation steps. J Pharm Biomed Anal. 2012;66:11–23. doi: 10.1016/j.jpba.2012.03.034 22494517

[18] Zhang S-D, Gant TW. Effect of pooling samples on the efficiency of comparative studies using microarrays. Bioinformatics. 2005;21(24):4378–83. doi: 10.1093/bioinformatics/bti717 16234321

[19] Yao L, Jiang Y, Singanusong R, D’Arcy B, Datta N, Caffin N, et al. Flavonoids in Australian Melaleuca, Guioa, Lophostemon, Banksia and Helianthus honeys and their potential for floral authentication. Food Res Int. 2004;37(2):166–74. doi: 10.1016/j.foodres.2003.11.004

[20] Soares S, Amaral JS, Oliveira MBPP, Mafra I. A Comprehensive Review on the Main Honey Authentication Issues: Production and Origin. Comprehensive Reviews in Food Science and Food Safety. 2017;16(5):1072–100. doi: 10.1111/1541-4337.12278 33371614

[21] Kortesniemi M, Rosenvald S, Laaksonen O, Vanag A, Ollikka T, Vene K, et al. Sensory and chemical profiles of Finnish honeys of different botanical origins and consumer preferences. Food Chem. 2018;246:351–9. doi: 10.1016/j.foodchem.2017.10.069 29291860

[22] Locher C, Neumann J, Sostaric T. Authentication of honeys of different floral origins via high-performance thin-layer chromatographic fingerprinting. JPC - Journal of Planar Chromatography - Modern TLC. 2017;30(1):57–62. doi: 10.1556/1006.2017.30.1.8

[23] Locher C, Tang E, Neumann J, Sostaric T. High-performance thin-layer chromatography profiling of Jarrah and Manuka honeys. JPC - Journal of Planar Chromatography - Modern TLC. 2018;31(3):181–9. doi: 10.1556/1006.2018.31.3.1

[24] Stanek N, Jasicka-Misiak I. HPTLC Phenolic Profiles as Useful Tools for the Authentication of Honey.(Report). Food Analytical Methods. 2018;11(11):2979. doi: 10.1007/s12161-018-1281-3

[25] Bouhlali EdT, Bammou M, Sellam K, El Midaoui A, Bourkhis B, Ennassir J, et al. Physicochemical properties of eleven monofloral honey samples produced in Morocco. Arab Journal of Basic and Applied Sciences. 2019;26(1):476–87.

[26] Burns DT, Dillon A, Warren J, Walker MJ. A Critical Review of the Factors Available for the Identification and Determination of Mānuka Honey. Food Analytical Methods. 2018;11(6):1561–7. doi: 10.1007/s12161-018-1154-9 Burns2018.

[27] Rodney S, Purdy J. Dietary requirements of individual nectar foragers, and colony-level pollen and nectar consumption: a review to support pesticide exposure assessment for honey bees. Apidologie. 2020;51(2):163–79. doi: 10.1007/s13592-019-00694-9 Rodney2020.

[28] Donkersley P, Rhodes G, Pickup RW, Jones KC, Power EF, Wright GA, et al. Nutritional composition of honey bee food stores vary with floral composition. Oecologia. 2017;185(4):749–61. doi: 10.1007/s00442-017-3968-3 29032464 PMC5681600

[29] Al ML, Daniel D, Moise A, Bobis O, Laslo L, Bogdanov S. Physico-chemical and bioactive properties of different floral origin honeys from Romania. Food Chem. 2009;112(4):863–7. doi: 10.1016/j.foodchem.2008.06.055

[30] Nayik GA, Nanda V. A chemometric approach to evaluate the phenolic compounds, antioxidant activity and mineral content of different unifloral honey types from Kashmir, India. LWT. 2016;74:504–13. doi: 10.1016/j.lwt.2016.08.016

[31] Bogdanov S, Haldimann M, Luginbühl W, Gallmann P. Minerals in honey: environmental, geographical and botanical aspects. J Apic Res. 2007;46(4):269–75. doi: 10.1080/00218839.2007.11101407

[32] Hingston FJ, Dimmock GM, Turton AG. Nutrient distribution in a jarrah (Eucalyptus marginata Donn ex Sm.) ecosystem in south-west Western Australia. For Ecol Manage. 1980;3:183–207. doi: 10.1016/0378-1127(80)90015-8

[33] Roshan N, Rippers T, Locher C, Hammer KA. Antibacterial activity and chemical characteristics of several Western Australian honeys compared to manuka honey and pasture honey. Arch Microbiol. 2017;199(2):347–55. doi: 10.1007/s00203-016-1308-3 Roshan2017. 27785532

[34] Tsagkaris AS, Koulis GA, Danezis GP, Martakos I, Dasenaki M, Georgiou CA, et al. Honey authenticity: analytical techniques, state of the art and challenges. RSC Advances. 2021;11(19):11273–94. doi: 10.1039/d1ra00069a PMC8695996 35423655

[35] Sniderman JMK, Matley KA, Haberle SG, Cantrill DJ. Pollen analysis of Australian honey. PLoS One. 2018;13(5):e0197545. doi: 10.1371/journal.pone.0197545 29768495 PMC5955576

[36] Li LN-LIRX. Pondering over and under pollen representation in nectar. New Zealand beekeeper. 2017;25(10):25–7.

[37] Molan PC. The limitations of the methods of identifying the floral source of honeys. Bee World. 1998;79(2):59–68. doi: 10.1080/0005772x.1998.11099381

[38] Robertson K. Understanding the value of pollen counting. New Zealand beekeeper. 2019;27(3):47–51.

[39] Stephens JM, Schlothauer RC, Morris BD, Yang D, Fearnley L, Greenwood DR, et al. Phenolic compounds and methylglyoxal in some New Zealand manuka and kanuka honeys. Food Chem. 2010;120(1):78–86. doi: 10.1016/j.foodchem.2009.09.074

[40] Makowicz E, Jasicka-Misiak I, Teper D, Kafarski P. HPTLC Fingerprinting-Rapid Method for the Differentiation of Honeys of Different Botanical Origin Based on the Composition of the Lipophilic Fractions. Molecules. 2018;23(7). Epub 2018/07/25. doi: 10.3390/molecules23071811 . 30037090 PMC6099833

[41] Stanek N, Kafarski P, Jasicka-Misiak I. Development of a high performance thin layer chromatography method for the rapid qualification and quantification of phenolic compounds and abscisic acid in honeys. J Chromatogr A. 2019;1598:209–15. Epub 2019/04/27. doi: 10.1016/j.chroma.2019.04.052 . 31023479

[42] Stanek N, Teper D, Kafarski P, Jasicka-Misiak I. Authentication of phacelia honeys (Phacelia tanacetifolia) based on a combination of HPLC and HPTLC analyses as well as spectrophotometric measurements. LWT. 2019;107:199–207. doi: 10.1016/j.lwt.2019.03.009

[43] Team RC. R: A Language and Environment for Statistical Computing. R Foundation for Statistical Computing; 2020.

[44] Team R. RStudio: Integrated Development Environment for R. Boston, MA: RStudio, PBC.; 2020.

